# Myrtenal Pretreatment Exerts a Protective Effect Against Renal Ischemia‐Reperfusion Injury in Rats

**DOI:** 10.1002/jbt.70202

**Published:** 2025-03-02

**Authors:** Leyla Beytur, Engin Korkmaz, Evren Köse, Aslı Taşlıdere, Suat Tekin

**Affiliations:** ^1^ Department of Anatomy, Faculty of Medicine Inonu University Malatya Turkey; ^2^ Department of Physiology, Faculty of Medicine Inonu University Malatya Turkey; ^3^ Department of Histology and Embryology, Faculty of Medicine Inonu University Malatya Turkey

**Keywords:** acute kidney injury, apoptosis, inflammation, myrtenal, oxidative stress

## Abstract

Acute kidney injury (AKI) is a serious condition with high mortality in intensive care units and during vascular surgeries. Renal ischemia‐reperfusion injury (IRI) significantly contributes to AKI. Preclinical studies have shown that myrtenal (Myrt) has anti‐inflammatory and antioxidant properties. We hypothesized that Myrt might alleviate renal damage caused by IRI through the modulation of oxidative stress and inflammatory processes. This study aimed to investigate the renoprotective potential of Myrt against IRI using biochemical evaluations and histological examinations. Forty male *Sprague Dawley* rats were assigned to four groups: sham, IRI, Myrt40+IRI, and Myrt80+IRI. Animals received daily intraperitoneal injections of Myrt (40–80 mg/kg) or solvent for 9 days before surgery. The sham group underwent laparotomy, while the other groups had AKI induced via bilateral renal pedicle clamping for 45 min, followed by 24 h of reperfusion. Renal function was assessed by blood urea nitrogen (BUN), creatinine, kidney injury molecule‐1 (KIM‐1) and neutrophil gelatinase‐associated lipocalin (NGAL) levels. Inflammatory markers interleukin‐1 beta (IL‐1β), tumor necrosis factor‐alpha (TNF‐α) were measured, along with oxidative stress parameters malondialdehyde (MDA), superoxide dismutase (SOD), catalase (CAT), and glutathione (GSH). Renal damage was evaluated histopathologically using hematoxylin and eosin staining and apoptosis was assessed via caspase‐3 immunohistochemistry. In the IRI group, serum BUN and creatinine levels were significantly higher than the sham group but were reduced by Myrt pretreatment (*p* < 0.05). IRI also led to significant increases in MDA, KIM‐1, NGAL, IL‐1β, and TNF‐α in kidney tissue (*p* < 0.05), which were notably decreased by Myrt pretreatment (*p* < 0.05). Myrt also restored SOD and CAT enzyme activities and GSH levels reduced by IRI (*p* < 0.05). Histological analysis showed Myrt significantly alleviated renal tissue damage and reduced caspase‐3 immunoreactivity due to IRI (*p* < 0.05). The findings suggest that Myrt has a protective effect against renal IRI.

## Graphical abstract

Myrtenal pretreatment has a protective effect against renal ischaemia‐reperfusion injury by reducing oxidative stress.

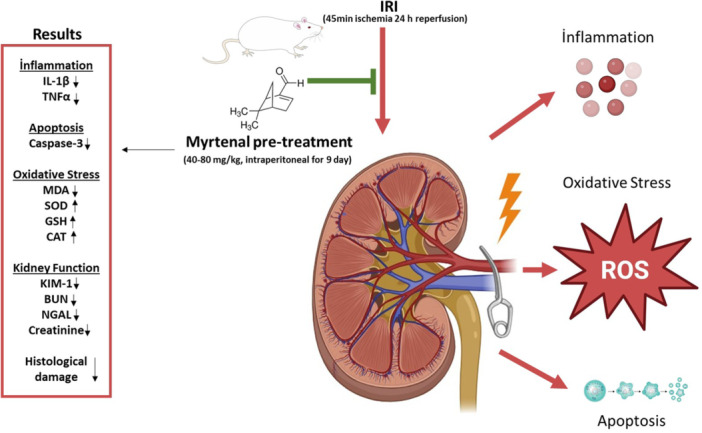

## 1 Introduction

Acute kidney injury (AKI) is a widespread health concern globally, affecting over 13 million people in developing countries every year [1]. In developed countries, the incidence of AKI is approximately 20% among hospitalized patients and ranges from 20% to 50% in those admitted to intensive care units [2, 3]. Renal ischemia‐reperfusion injury (IRI) is recognized as a significant cause of AKI [4, 5]. The pathophysiology of IRI is complex; however, it is known that oxidative stress triggers harmful cascades, including apoptosis and inflammation [6]. During renal IRI, the production of reactive oxygen species (ROS) increases, leading to cellular damage and the release of malondialdehyde (MDA), a product of lipid peroxidation [7]. To protect against ROS, renal cells actively release antioxidant enzymes such as glutathione peroxidase (GPx), catalase (CAT), and superoxide dismutase (SOD). Nevertheless, the ability of these antioxidants to combat damage diminishes during renal IRI. The subsequent imbalance between oxidants and antioxidants and ensuing DNA damage promote the apoptotic process [8]. In this process, the activity of caspases which are key indicators of renal IRI is heightened [9]. Concurrently, ROS formation and an increase in necrotic cells in renal tissue trigger a cascade that activates pro‐inflammatory cytokines [10]. Following renal injury, activated macrophages release pro‐inflammatory cytokines such as TNF‐α, IL‐1β, and IL‐6 [11]. These cytokines enhance inflammation by facilitating the infiltration of neutrophils, monocytes and T cells into the damaged area, thus exacerbating tissue damage [12]. The lack of effective treatments or preventive strategies has contributed to AKI remaining a critical health issue [13]. Therefore, developing therapeutic strategies aimed at reducing oxidative stress, apoptosis, and inflammation could be a pivotal step in managing AKI.

Natural products and their molecules play a crucial role in drug discovery and development. Over the past three decades, around 50% of the drugs approved have either been directly derived from natural products or contain components based on them [14]. Research aimed at developing strategies to combat IRI in various organs often investigates the potential of natural antioxidant and anti‐inflammatory compounds [15, 16, 17]. Monoterpenes, which are found in the essential oils of many plants, have shown promising pharmacological activity against renal IRI, primarily through their antioxidant properties [18, 19, 20]. Myrtenal (Myrt), a monoterpene found in the essential oils of plants such as eucalyptus, mint, pepper, and cumin [21], has demonstrated antioxidative and anti‐inflammatory effects in experimental disease models. In particular, studies have shown that it increases the activity of endogenous antioxidant systems such as CAT, SOD, and GPx in the liver and pancreas of diabetic rats [22], as well as in the brain tissue of mice with dementia, while reducing levels of oxidative‐inflammatory markers like TNF‐α and MDA [23]. However, to the best of our knowledge, there is currently no data on the effects of Myrt on renal IRI. This study aims to investigate whether Myrt pretreatment can provide a protective effect against renal IRI.

## 2 Materials and Methods

### 2.1 Animals

This study was conducted with the approval of the Inonu University Faculty of Medicine Animal Experiments Local Ethics Committee (2023/2‐1). The experimental model and analyses were carried out in the laboratories of the Experimental Animal Production Research Center, the Departments of Physiology and Histology and Embryology at Inonu University Faculty of Medicine. The number of animals used in the experiments was determined through a power analysis, based on an estimated initial body weight range of 260–290 g, a standard deviation of 15 g, a 4% margin of error, with a type 1 error (*α*) of 0.05 and a type 2 error (*β*) of 0.80. This analysis indicated that at least 10 animals were required in each group. Before starting the experiment, the animals were weighed and randomly assigned to groups according to their body weight using a computer‐based randomization algorithm (MedCalc 12.7.0 for Windows). A one‐way ANOVA revealed no significant differences in body weight between the groups (*p* = 0.253).

Forty male *Sprague‐Dawley* rats, weighing 235–293 g, were used in the study. The animals were housed under a 12‐h light/dark cycle at a controlled temperature of 22 ± 2°C and 50 ± 10% humidity. They were fed standard rodent chow ad libitum and had free access to water.

### 2.2 Experiment Design

**Figure 1:**
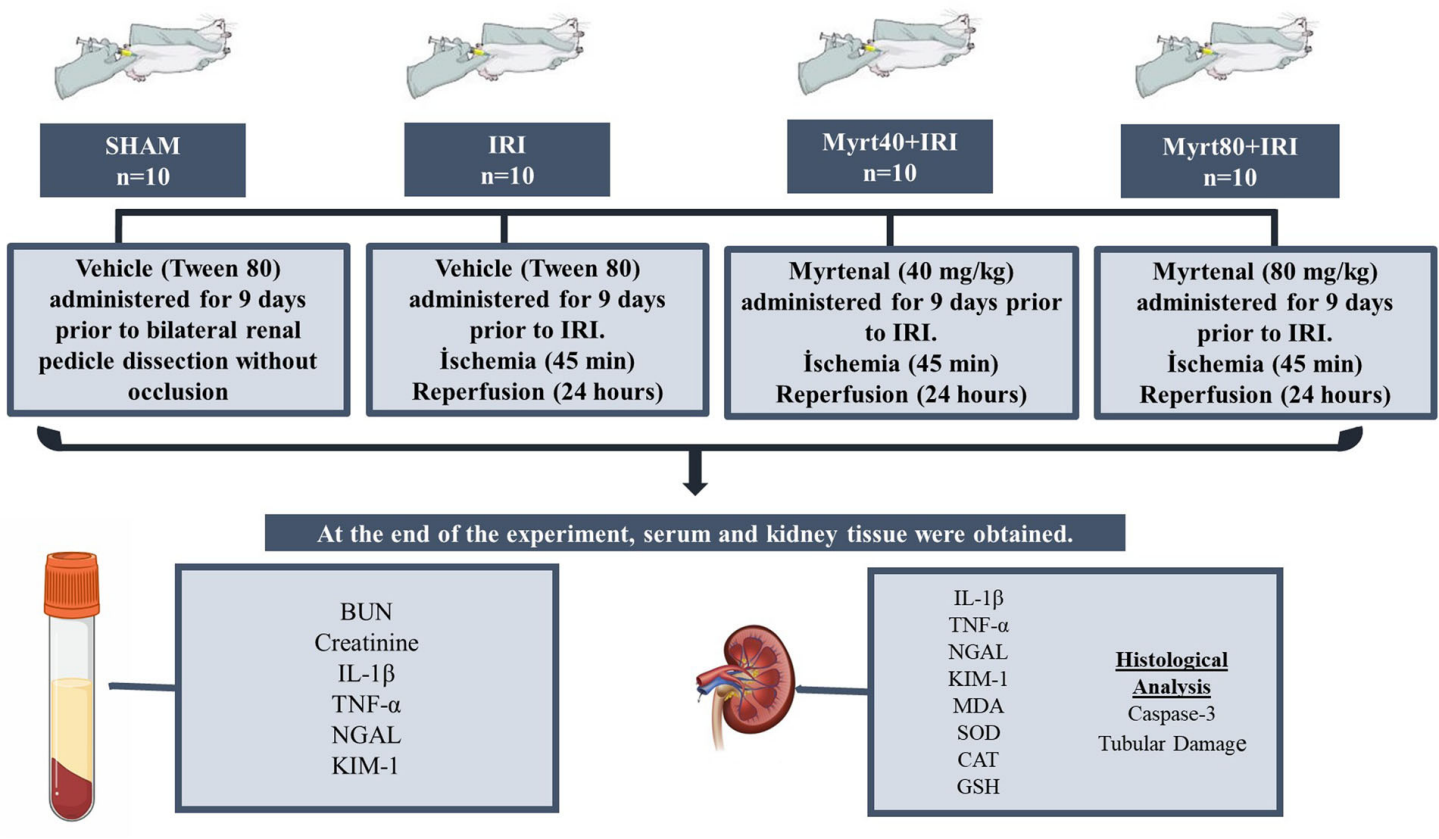
Experimental design of the study. BUN, blood urea nitrogen; CAT, catalase; GSH, glutathione; IL‐1β, interleukin‐1 beta; IRI, ischemia reperfusion injury; KIM‐1, kidney injury molecule‐1; MDA, malondialdehyde Myrt, myrtenal; NGAL, neutrophil gelatinase‐associated lipocalin; SOD, superoxide dismutase; TNF‐α, tumor necrosis factor‐alpha.

The rats were divided into four groups (*n* = 10, per group): Sham, IRI, Myrt40+IRI, and Myrt80+IRI. For 9 days before surgery, the Sham and IRI groups were administered Vehicle (Tween80, 0.1%), while the Myrt40+IRI and Myrt80+IRI groups received Myrt at doses of 40 and 80 mg/kg respectively, via intraperitoneal injection. The dosage and administration schedule for Myrt were based on prior studies [24, 25]. Myrt (Sigma Aldrich, Cat#218243, USA) was dissolved in Tween80 (0.1%) [26] for administration. Following pretreatment with Myrt, the animals were anesthetized using a combination of 10 mg/kg xylazine and 100 mg/kg ketamine. IRI was then induced according to the method described by Malek and Nematbakhsh [27]. Bilateral incisions were made to expose the kidneys and the renal pedicles (comprising the artery, vein and nerve) were clamped for 45 min. The occlusion was visually confirmed by the pale color of the kidneys and reperfusion was verified by the reddening of the kidneys. Animals that did not exhibit reperfusion were excluded from the study. Sham group underwent all procedures except for occlusion. After 24 h of reperfusion, the animals were decapitated and blood and kidney tissues were collected for analysis. Blood samples were centrifuged to obtain serum and both the serum and kidney tissue samples were stored at −80°C until further analysis. The experimental plan is illustrated in Figure 1.

## 3 Biochemical Analyses

### 3.1 Tissue Preparation

Kidney tissues stored at −80°C were retrieved for analysis. During the preparation of the tissue homogenates, phosphate‐buffered saline (PBS) (0.01 M, pH = 7.4) was used to wash away hemolyzed blood to prevent interference with the test results. After the tissue weights were recorded, the samples were placed in homogenizer tubes. The homogenates were then centrifuged at 5000*g* for 5 min at +4°C to separate the supernatant. The total protein concentration in the supernatant was determined using a commercial BCA kit (ABP Biosciences, Cat#P011, USA). Blood samples were centrifuged at 5000 rpm for 10 min to separate the serum.

### 3.2 Evaluation of Renal Function, Damage Markers, and Inflammatory Parameters

Blood urea nitrogen (BUN) and creatinine levels in serum samples were measured using an automated biochemical analyzer (Roche Diagnostics, Mannheim, Germany). Serum and renal tissue levels of neutrophil gelatinase‐associated lipocalin (NGAL) (SunRed, Cat#201‐11‐0763, China), kidney injury molecule‐1 (KIM‐1) (SunRed, Cat#201‐11‐0550, China), interleukin‐1 beta (IL‐1β) (SunRed, Cat#201‐11‐0120, China), and tumor necrosis factor‐alpha (TNF‐α) (SunRed, Cat#201‐11‐0765, China) were measured using commercial ELISA kits, following the manufacturer's instructions.

### 3.3 Evaluation of Oxidative Stress Markers in the Kidney

MDA analysis was performed according to the method described by Esterbauer and Cheeseman [28]. MDA reacts with thiobarbituric acid (TBA), producing a pink pigment with a maximum absorbance at 532 nm. The values for each sample were derived from a standard curve and expressed as nmol/mg protein. The concentration of GSH was determined using Beutler's method [29], with total GSH levels calculated from a standard curve generated using known quantities of GSH. GSH levels were expressed as µmol/mg protein. CAT enzyme activity was determined following Aebi's method [30], measuring the rate constant k (dimension: s‐1) of hydrogen peroxide decomposition at 240 nm. The results were expressed as U/mg protein. SOD enzyme activity was measured using the method described by McCord and colleagues and reported as U/mg protein [31].

### 3.4 Histopathological and Immunohistochemical Examinations

Kidney samples were fixed in 10% formalin for light microscopic evaluation, processed using standard tissue techniques and embedded in paraffin. The paraffin‐embedded samples were sectioned into 5 mm slices, mounted on slides and stained with Hematoxylin‐Eosin (H‐E). The tissue samples were examined using a light microscope (Leica DFC280) and analyzed with a Leica Q Win Image Analysis system (Leica Micros Imaging Solutions Ltd., Cambridge, UK).

Histopathological evaluation assessed tissue damage using various parameters, including tubular lumen dilation, hemorrhage and inflammatory cell infiltration, hydropic degeneration, hemorrhage between tubules and glomeruli, epithelial atrophy and cell desquamation in the tubules, vacuolization in tubular epithelial cells, and debris in the tubular lumen. Each sample was scored semiquantitatively using a scale from 0 to 3 (0 = none, 1 = mild, 2 = moderate, 3 = severe) for each criterion and the total scores were calculated based on these parameters [32]. Microscopic scoring and cell counting were performed blindly by the same histologist.

For immunohistochemical (IHC) analysis, sections were mounted on poly‐L‐lysine‐coated slides. After rehydration, the samples were transferred to citrate buffer (pH 7.6) and heated in a microwave. After cooling to room temperature, the sections were washed with PBS. The samples were then incubated in 0.3% hydrogen peroxide, washed with PBS, and incubated with primary rabbit polyclonal caspase‐3 antibody (Boster, Cat#PA1302, USA) for 2 h. After washing in PBS, the sections were incubated with biotinylated goat antipolyvalent and streptavidin peroxidase at room temperature. Staining was completed using chromogen and substrate, followed by counterstaining with Mayer's hematoxylin. The slides were then rinsed in tap water and dehydrated. Caspase‐3 was used according to the manufacturer's instructions [33] and caspase‐3‐positive cells appeared brown. In caspase‐3 positive staining scoring, staining intensity was evaluated as follows: 0 for 0%–25%, 1 for 25%–50%, 2 for 50%–75%, and 3 for 75%–100%.

### 3.5 Statistical Analysis

IBM SPSS Statistics 24.0 for Windows was used for the statistical analysis of the data. Quantitative data were summarized as mean ± standard deviation (SD). The normality of the data distribution was assessed using the Shapiro–Wilk test. The Kruskal–Wallis H test was used for comparisons of quantitative variables between groups. If significant differences were identified, multiple comparisons were made using the Bonferroni‐adjusted Mann–Whitney *U* test. A *p* < 0.05 was considered statistically significant.

## 4 Results

**Figure 2:**
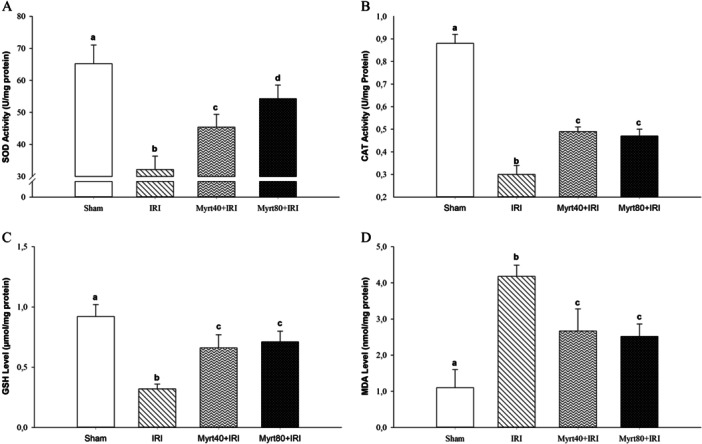
Effect of Myrt on SOD (A) and CAT activity (B), GSH (C), and MDA (D) levels. (Evaluation of the data was performed using the Kruskal–Wallis H test. Multiple comparisons were made using the Bonferroni corrected the Mann–Whitney *U* test. Values are expressed as mean ± SD. Different letters indicate statistical difference between groups; ^a, b, c, d^ *p* < 0.05). CAT, catalase; GSH, glutathione; MDA, malondialdehyde; SOD, superoxide dismutase.

The levels of GSH, CAT, and SOD in kidney tissue were significantly higher in the Myrt‐treated group compared to the IRI group. The increases in GSH and CAT levels were dose‐independent, while SOD levels showed a dose‐dependent increase (Figure 2A–C; *p *< 0.05). On the other hand, the MDA levels in kidney tissue were significantly higher in the IRI group compared to the other groups (*p* < 0.05). However, the MDA levels in the Myrt‐treated groups were significantly lower than those in the IRI group (Figure 2D; *p* < 0.05). These effects were similar in both the low‐ and high‐dose Myrt groups.

**Figure 3:**
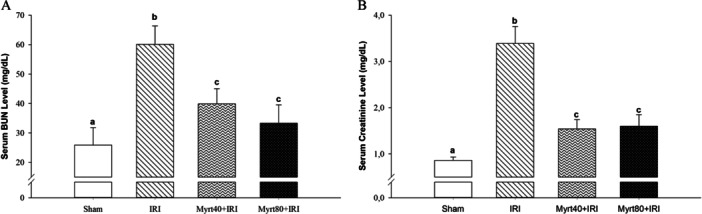
Effect of Myrt on serum BUN (A) and serum creatinine (B) levels. (Data were analyzed using the Kruskal–Wallis H test. Multiple comparisons were performed using the Bonferroni corrected Mann–Whitney *U* test. Values are expressed as mean ± SD. Different letters indicate statistical difference between groups; ^a, b, c^ *p* < 0.05). BUN, blood urea nitrogen.

The serum BUN and creatinine levels of the groups are shown in Figure 3. Serum BUN and creatinine levels in the IRI group were significantly higher than those in the sham group (*p *< 0.05). In contrast, the Myrt‐treated groups showed a marked reduction in serum BUN and creatinine levels compared to the IRI group (*p *< 0.05; Figure 3A,B).

**Figure 4:**
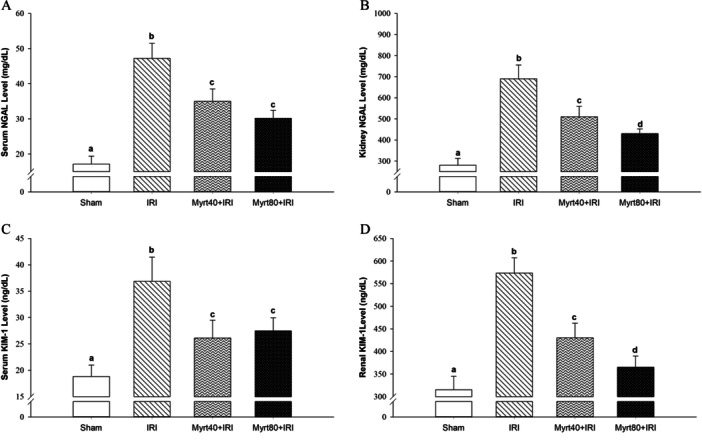
Serum NGAL (A) and renal NGAL (B) levels and its effect on serum KIM‐1 (C), and renal KIM‐1 (D) levels. (Evaluation of the data was performed using the Kruskal–Wallis H test. Multiple comparisons were made using the Bonferroni corrected the Mann–Whitney *U* test. Values are expressed as mean ± SD. Different letters indicate statistical difference between groups; ^a, b, c, d^ *p* < 0.05). KIM‐1, kidney injury molecule‐1; NGAL, neutrophil gelatinase‐associated lipocalin.

Serum and renal levels of NGAL and KIM‐1 in the groups are presented in Figure 4. Comparisons of serum NGAL levels across the experimental groups revealed that both serum and renal NGAL and KIM‐1 levels were significantly elevated in the IRI group compared to the sham group (*p *< 0.05). However, in the Myrt‐treated groups (40 and 80 mg/kg), serum NGAL and KIM‐1 levels were dose‐independent, while renal NGAL and KIM‐1 levels were dose‐dependent, with all levels significantly lower than those in the IRI group (*p *< 0.05; Figure 4A–D).

**Figure 5:**
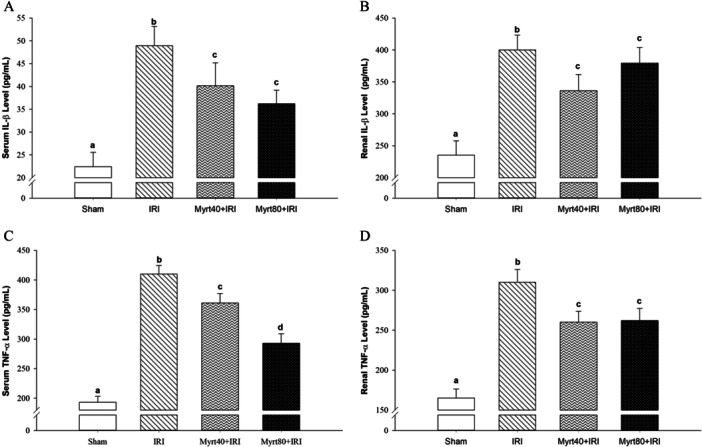
Effect of Myrt on serum IL‐1β (A) and renal IL‐1β (B) levels and its effect on serum TNF‐α (C), and renal TNF‐α (D) levels (Evaluation of the data was performed using the Kruskal–Wallis H test. Multiple comparisons were made using the Bonferroni corrected the Mann–Whitney *U* test. Values are expressed as mean ± SD. Different letters indicate statistical difference between groups; ^a, b, c, d^ *p* < 0.05). IL‐1β, interleukin‐1 beta; TNF‐α, tumor necrosis factor‐alpha.

The effects of Myrt treatment on IL‐1β and TNF‐α levels in both serum and renal tissue across the groups are shown in Figure 5. Comparisons of serum IL‐1β and TNF‐α levels revealed that both serum and renal TNF‐α and IL‐1β levels were significantly elevated in the IRI group compared to the sham group (*p *< 0.05). In the Myrt‐treated groups, TNF‐α and IL‐1β levels were reduced compared to the IRI group. This effect was dose‐independent, except for the serum TNF‐α levels (*p *< 0.05; Figure 5A–D).

**Figure 6:**
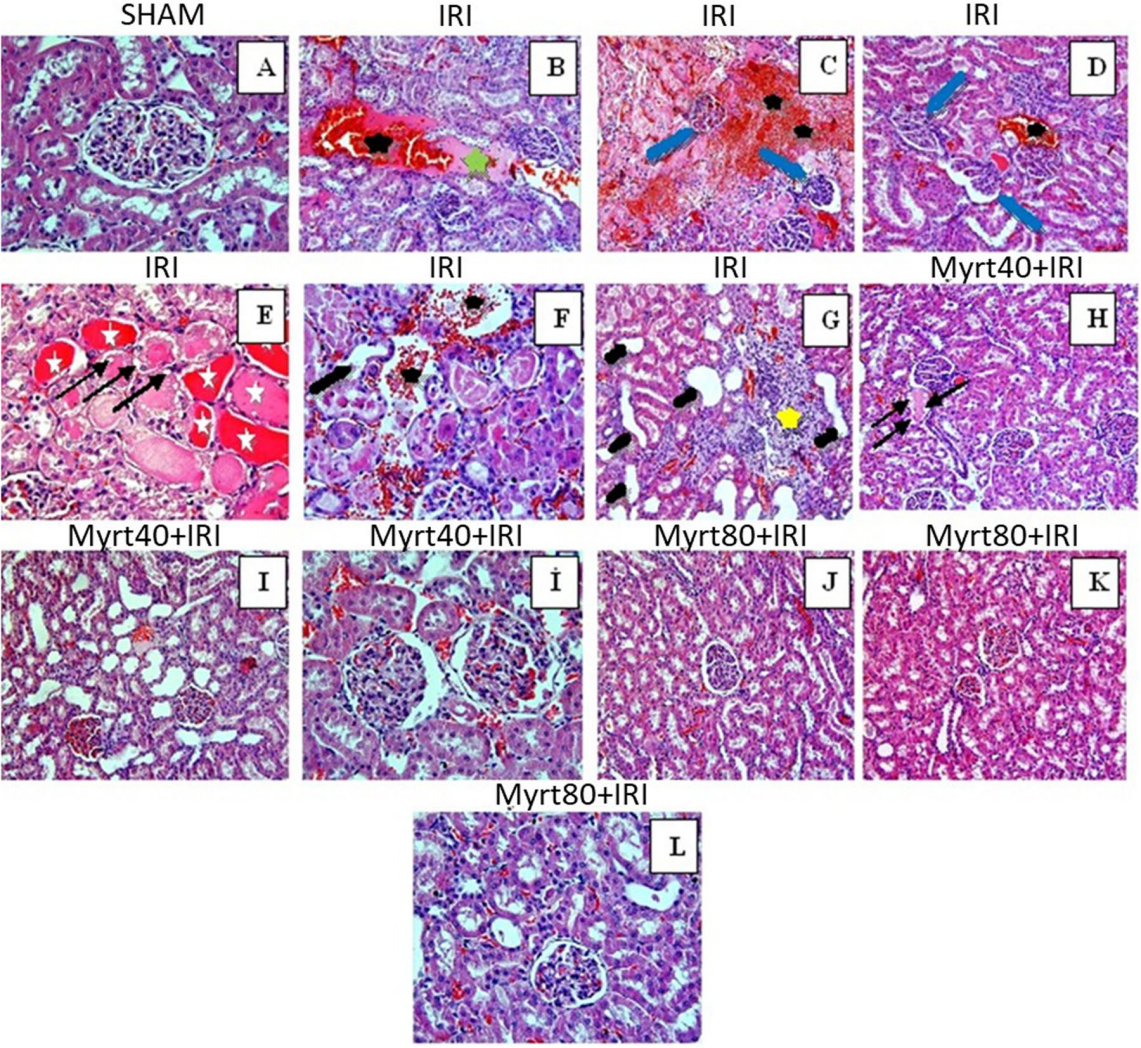
Histological evaluation of kidney sections in all groups. In the sham group (A), glomerular and tubular structures were normal. In the IRI group (B–G), severe histopathological damage findings such as hemorrhage (black star), edema (green star), glomerular degeneration (blue arrow), tubular lumen dilation (thick black arrows), epithelial atrophy, and cell detachment in the tubules (white star and vacuolization of tubular epithelial cells (thin black arrows), inflammatory cell infiltration (yellow star) were observed. In Myrt40+IRI group (H and i), a small amount of hemorrhage, vacuolization, edema, and tubular dilatation were observed. Some haemorrhage and tubular dilatation were detected in Myrt80+IRI group (J–L). (B–D, J, K, H, I): H&E X20; (A, E, F, İ, L): H&E; X40.

Histological examination revealed a normal kidney structure in the sham group (Figure 6A). In contrast, the IRI group exhibited histopathological changes and significant renal injury. These changes included hemorrhage (black stars; Figure 6B–D,F), glomerular degeneration (Figure 6C,D; blue arrow), tubular lumen dilation (thick black arrows; Figure 6F,G), inflammatory cell infiltration (yellow stars; Figure 6G), and edema (green stars; Figure 6B). Additionally, epithelial atrophy and cell detachment within the tubules (Figure 6C,E,F,G), vacuolization of tubular epithelial cells (thin black arrows; Figure 6E) and debris in the tubular lumen (Figure 6D,E) were observed. Compared to the IRI group, the Myrt40+IRI group and the Myrt80+IRI group displayed significantly improved histological appearances. In the low‐dose Myrt40+IRI group, there was minimal hemorrhage (Figure 6H,I), vacuolization (thin black arrows; Figure 6H), edema (Figure 6H,I), and tubular dilation (Figure 6H,I). Meanwhile, the Myrt80+IRI group exhibited some hemorrhage and tubular dilation (Figure 6J–L). The kidney scores for all groups are summarized in Table 1.

**Table 1:** Histopathological damage scoring of the groups (different letters indicate statistical difference between groups; ^a, b, c, d^ *p* < 0.05).

Groups	Histopathological damage (mean ± SD)
Sham	0.43 ± 0.08^a^
IRI	2.23 ± 0.10^b^
Myrt40+IRI	1.50 ± 0.13^c^
Myrt80+IRI	1.17 ± 0.07^d^

**Figure 7:**
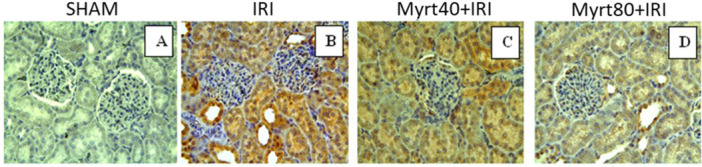
Immunohistochemical staining of Caspase‐3 in kidney sections. Sham (A), IRI group (B), Myrt40+IRI (C), Myrt80+IRI (D). Immunohistochemical staining shows a significant increase in the number of Caspase‐3 positive cells in the IRI group (B) compared to the sham group (A). The proportion of Caspase‐3 positive stained cells was minimal in the Myrt40+IRI group (C) and Myrt80+IRI group (D) compared to the IRI group. In the Myrt80+IRI group, the number of positive stained cells was significantly lower compared to the Myrt40+IRI group. (A–D): Caspase‐3; X40.

Immunohistochemical analysis showed a significant increase in the number of Caspase‐3 positive cells in the IRI group compared to the sham group (Figure 7A,B). The proportion of Caspase‐3 positive stained cells was minimal in the Myrt40+IRI (Figure 7C) and Myrt80+IRI groups (Figure 7D) compared to the IRI group. Furthermore, when comparing the Myrt80+IRI group with the Myrt40+IRI group, the Myrt80+IRI group had a significantly lower number of Caspase‐3 positive stained cells than the Myrt40+IRI group. The scores for the intensity of caspase‐3 positive staining are shown in Table 2.

**Table 2:** Intensity caspase‐3 positive staining score (different letters indicate statistical difference between groups; ^a, b, c, d^ *p* ≤ 0.0001).

Groups	Caspase‐3 positive staining score (mean ± SD)
Sham	0.33 ± 0.11^a^
IRI	2.05 ± 0.13^b^
Myrt40+IRI	1.48 ± 0.19^c^
Myrt80+IRI	1.14 ± 0.10^d^

## 5 Discussion

Renal IRI is widely recognized as a common cause of AKI and is notably associated with significant morbidity and mortality in intensive care patients and those undergoing major surgery [34, 35]. It is characterized by a sudden reduction in blood flow to the kidneys, followed by the reperfusion of oxygenated blood. This process results in a series of pathological damages in the glomeruli and tubules [36, 37]. These injuries, especially when they lead to renal dysfunction, can cause elevated serum BUN and creatinine levels [38]. Clinically, an increase in serum creatinine is traditionally used as a marker for diagnosing AKI [39]. However, while creatinine remains a standard marker for assessing renal function, more sensitive biomarkers such as KIM‐1 and NGAL reflect damage to renal tubular cells more accurately, enabling the early detection of AKI. Although the clinical use and evaluation of these biomarkers are still in progress, they are increasingly important for the rapid and effective identification of kidney injury [40]. In this study, increased levels of BUN, creatinine, KIM‐1, and NGAL in the IRI groups compared to the sham group clearly indicate the onset of renal IRI. Myrt pretreatment improved renal glomerular filtration, as demonstrated by the reduction in serum creatinine, BUN, KIM‐1, and NGAL levels. These findings support the consideration of Myrt as a potential renoprotective agent.

To understand the renoprotective properties of Myrt, focusing on the underlying pathophysiological mechanisms of renal IRI is essential. A key factor in the pathophysiology of renal IRI is the release of ROS. Elevated ROS levels cause cellular damage, leading to the release of MDA, a byproduct of lipid peroxidation [41, 42]. Cellular structures most vulnerable to ROS‐induced damage include proteins, lipids and nucleic acids, particularly DNA molecules [43]. Cells release endogenous antioxidant enzymes, such as GPx, CAT, and SOD, to counter ROS‐induced damage [44]. However, during IRI, the ability of antioxidants to counteract damage diminishes. This shift in the oxidant‐antioxidant balance toward oxidants due to IRI results in increased cellular injury [45]. In this study, Myrt pretreatment led to an increase in SOD and CAT enzyme activities and GSH levels in kidney tissue, resulting in decreased lipid peroxidation (MDA). Previous studies showing Myrt's ability to protect the brain from oxidative stress by maintaining the endogenous antioxidant system (GSH, CAT, SOD) in models of dementia in mice [23] and Parkinson's disease in rats [46], and its reduction of MDA levels in a bladder cancer model [47], are consistent with our findings. The cellular effects of Myrt and its chemical structure may play a significant role in the effects observed in this study and in the literature. As a lipid molecule, Myrt may support the structural integrity and function of cell or organelle membranes due to its membrane‐stabilizing properties. This can contribute to the construction and maintenance of robust and functional membranes [47, 48]. Additionally, bicyclic monoterpenoids like Myrt can react with ROS by their double bonds, neutralizing these harmful molecules. This property may help prevent or reduce the damage caused by free radicals to cells [49, 50, 51].

Increased ROS production following renal IRI results in a higher number of necrotic cells and the release of pro‐inflammatory cytokines, such as TNF‐α and IL‐1β [52]. These cytokines facilitate the infiltration of neutrophils, monocytes and T cells into the damaged area, contributing to tubular cell inflammation and exacerbating kidney injury [53]. Therefore, compounds with anti‐inflammatory properties that target cytokines like TNF‐α and IL‐1β are known to reduce kidney damage due to IRI [54]. In the current study, pretreatment with Myrt reduced TNF‐α and IL‐1β levels in both serum and kidney tissue. This reduction may be attributed to the immunomodulatory properties of Myrt against renal IRI. This finding suggests that Myrt has anti‐inflammatory activity in addition to its antioxidant effects. Supporting this, previous studies have shown that Myrt application suppresses increased TNF‐α and IL‐1β activation in vitro [55]. Additionally, it has been reported that Myrt administration reduces elevated levels of NF‐κB, TNF‐α, and IL‐1β in liver and pancreatic tissues in a rat model of Type 2 Diabetes Mellitus [22]. In a study on demented mice, Myrt treatment protected the brain from oxido‐inflammatory stress, thereby improving spatial memory deficits and reducing anxiety [23]. Although we were unable to measure NF‐κB levels in our study, the observed reduction in downstream TNF‐α and IL‐1β levels following Myrt administration is consistent with the literature. Our results further support that Myrt has anti‐inflammatory activity in addition to its antioxidant effects. These findings suggest that Myrt can effectively suppress inflammation associated with renal IRI.

An oxidant‐antioxidant imbalance in renal IRI can lead to DNA damage and the initiation of the apoptotic process [6]. Caspases are key regulators of apoptosis [56]. DNA fragmentation promotes the proteolytic activation of “executioner” caspases, such as caspase‐3, triggering apoptotic changes and marking the onset of an irreversible phase of programmed cell death [57]. We confirmed the presence of apoptosis in renal tubular cells by detecting caspase‐3 activation through immunohistochemical methods. In the Myrt‐treated groups, the number of caspase‐3 positive‐stained cells in kidney tissue was significantly reduced in a dose‐dependent manner compared to the IRI group. This finding aligns with previous studies indicating that Myrt has an apoptosis‐reducing effect on apoptotic parameters [58].

Renal IRI injury causes changes in renal blood flow, leading to microvascular damage and alterations in the reactive properties of affected renal vessels. This process is associated with damage to glomerular and tubular epithelial cells, inflammatory cell infiltration and apoptosis [59]. Our study showed significant histopathological changes and severe injuries in the kidney sections of animals in the IRI group. These changes included marked hemorrhage among glomeruli, dilation of the tubular lumen, inflammatory cell infiltration, edema, epithelial atrophy in the tubules, cell shedding, and vacuolization of tubular epithelial cells. Following both low and high‐dose Myrt administration, there was a significant reduction in kidney damage in rats compared to the IRI group. A significant difference was found between high‐ and low‐dose Myrt regarding the reduction of histological damage. In plants, Myrt is metabolically related to α‐pinene, which is metabolized into myrtenol and then converted into Myrt. Supporting our findings, these Myrt‐associated compounds have been reported to have beneficial effects on histopathological kidney changes [60, 61].

In summary, our consistent biochemical and histopathological findings demonstrate that Myrt effectively modulates oxidative stress to reduce AKI induced by renal IRI. These protective effects are likely achieved through the following mechanisms:

1. Enhancement of enzymatic and nonenzymatic antioxidants,
2. Decrease in lipid peroxidation,
3. Reduction in levels of pro‐inflammatory markers like TNF‐α and IL‐1β,
4. Lowering of apoptotic parameters such as caspase‐3.

## 6 Conclusion

**Figure 8:**
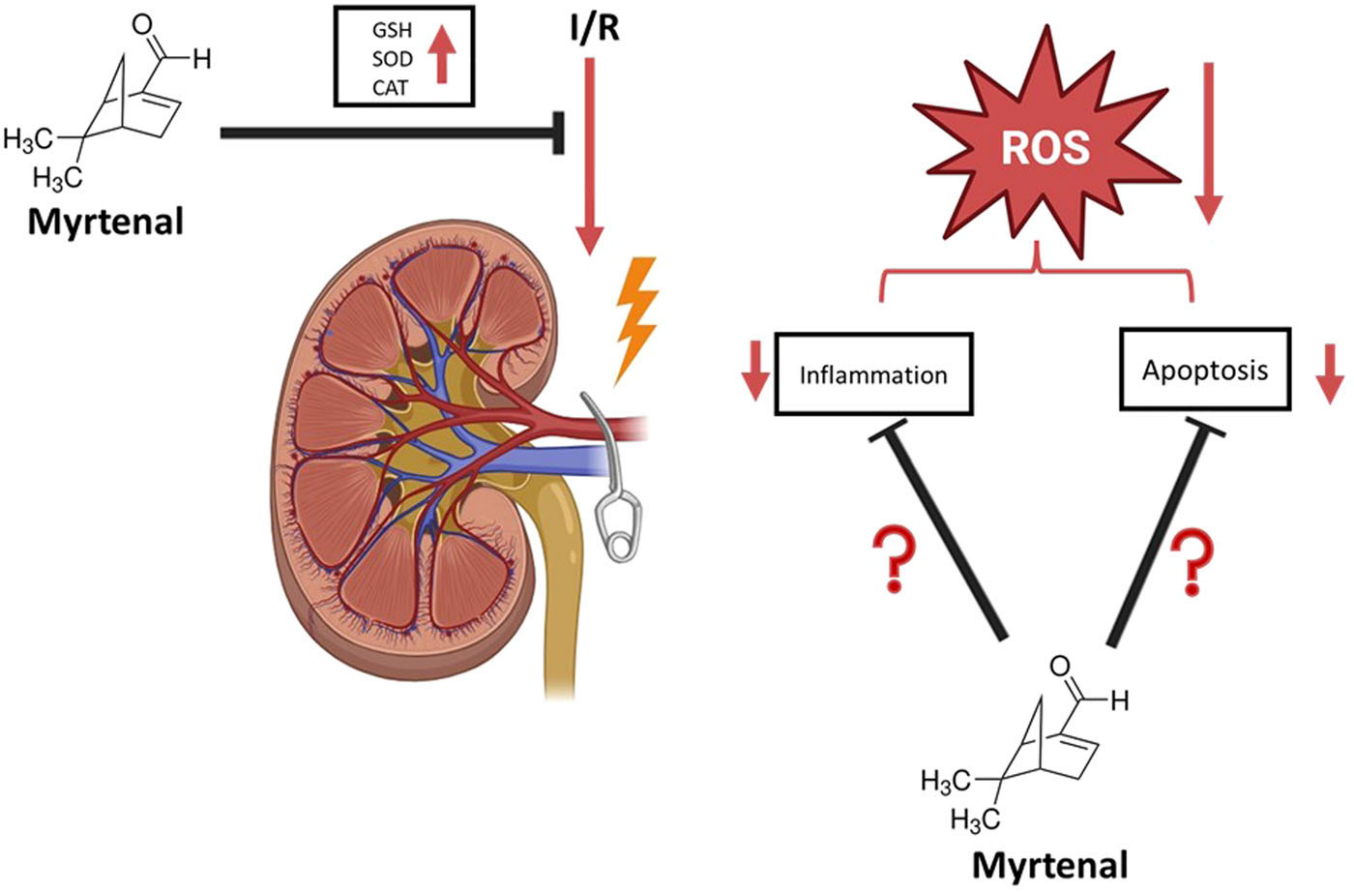
Effects observed and limitations in the study.

In conclusion, our study evaluating the potential effects of Myrt on renal IRI supports our hypothesis that Myrt may offer renoprotective potential associated with its antioxidant properties. However, the scope of this study does not provide clear information on whether the effects of Myrt are solely attributable to its antioxidant properties or if other mechanisms are involved in reducing apoptosis and inflammation. At this point, the gaps indicated by question marks in Figure 8 need to be addressed through advanced studies to elucidate the molecular mechanisms of Myrt's effects.

## Author Contributions

**Leyla Beytur:** writing – original draft, methodology, investigation. **Engin Korkmaz:** writing – original draft, methodology, investigation. **Suat Tekin:** writing – review and editing, methodology. **Aslı Taşlıdere:** methodology. **Evren Köse:** writing – review and editing, methodology, investigation. All authors have read and agreed to the published version of the manuscript.

## Ethics Statement

This study was conducted with the approval of the Inonu University Faculty of Medicine Animal Experiments Local Ethics Committee (2023/2‐1).

## Conflicts of Interest

The authors declare no conflicts of interest.

## Data Availability

The data generated in the present study are available upon request from the corresponding author.
